# Results of a survey among GP practices on how they manage patient safety aspects related to point-of-care testing in every day practice

**DOI:** 10.1186/s12875-014-0217-2

**Published:** 2015-02-05

**Authors:** Claudette de Vries, Carine Doggen, Ellen Hilbers, Robert Verheij, Maarten IJzerman, Robert Geertsma, Ron Kusters

**Affiliations:** Centre for Health Protection, National Institute for Public Health and the Environment (RIVM), P.O. Box 1, NL-3720 BA Bilthoven, The Netherlands; Health Technology and Services Research, MIRA institute for Biomedical Technology and Technical Medicine, University of Twente, Enschede, The Netherlands; Netherlands institute for health services research, Utrecht, The Netherlands; Clinical Chemistry and Haematology laboratory, Jeroen Bosch Ziekenhuis, Utrecht, The Netherlands

## Abstract

**Background:**

Point-of-care (POC) tests are devices or test strips that can be used near or at the site where care is delivered to patients, enabling a relatively fast diagnosis. Although many general practitioners (GPs) in the Netherlands are using POC tests in their practice, little is known on how they manage the corresponding patient safety aspects.

**Methods:**

To obtain information on this aspect, an invitation to participate in a web-based questionnaire was sent to a random sample of 750 GP practices. Of this sample 111 GP practices returned a complete questionnaire. Data was analysed by using descriptive statistics.

**Results:**

Results show that there is not always attention for quality control measures such as checking storage conditions, executing calibration, and maintenance. In addition, universal hygienic measures, such as washing hands before taking a blood sample, are not always followed. Refresher courses on the use of POC tests are hardly organized. Only a few of the GPs contact the manufacturer of the device when a device failure occurs. Well-controlled aspects include patient identification and actions taken when ambiguous test results are obtained.

**Conclusions:**

We observed a number of risks for errors with POC tests in GP practices that may be reduced by proper training of personnel, introduction of standard operating procedures and measures for quality control and improved hygiene. To encourage proper use of POCT in general practices, a national POCT guideline, dedicated to primary care and in line with ISO standards, should be introduced.

**Electronic supplementary material:**

The online version of this article (doi:10.1186/s12875-014-0217-2) contains supplementary material, which is available to authorized users.

## Background

Health care professionals are using various types of point-of-care (POC) tests for therapeutic decision-making [1]. The main advantage of using POC tests is that results can be obtained very rapidly at or near the site where care is delivered to the patient. These diagnostic tests enable general practitioners (GPs) to make a clinical decision during the patient’s visit. The use of POC tests reduces the number of referrals to a central laboratory and may also result in reducing the need for a second patient visit. This is convenient for patients and may enhance practice efficiency.

Although instant diagnostic information obtained through POC tests may increase quality and efficiency of care delivery, it also carries a risk for the patient if the result is false-positive or false-negative [2]. Various non-laboratory health care professionals, who usually do not have the adequate training and experience to sample and analyse human body materials, use POC tests in different settings. In the Netherlands GPs have guidelines for the diagnoses of various diseases, in which diagnostic tools, such as POC tests, are described. However, quality requirements specifically for the use of POC test in GP practices in the Netherlands are missing. Results of a study among practice nurses in the UK showed that they have a poor understanding of quality control issues related to near-patient testing, e.g. of maintenance of equipment and management of the test results [3]. In 2007, the Netherlands Society for Clinical Chemistry and Laboratory Medicine (NVKC) analysed reports on serious incidents after the use of blood glucose meters in various Dutch hospitals. The NVKC concluded that in most cases the incorrect use of the blood glucose meters was the underlying cause for these incidents [4]. These incidents emphasized the need for quality requirements for the use of POC tests.

To mitigate the risks of POC testing (POCT) and to ensure patient safety, a health care professional has to adopt a systematic approach when using POC tests. In literature, several indicators are described for managing POC tests-related patient safety aspects [4-10], such as training, universal hygienic measures, quality control measures, registration of results, and established alternative actions when test results conflict with symptoms or when device failure occurs.

However, little is known on how GP practices in the Netherlands manage safety aspects related to POCT in every day practice. This study is the first study in the Netherlands to systematically evaluate safety aspects of POCT in GP practices.

## Methods

### Questionnaire

Results of a previous survey among GP practices on POCT in 2010 showed that tests to measure nitrite to identify a urinary infection, blood glucose to determine the blood glucose levels and haemoglobin to identify anaemia, are used most frequently by GPs in the Netherlands [11]. Therefore, questions related to these three tests, more specifically held hand devices, were included in this study. To obtain information on the management of patient safety aspects of POCT, a questionnaire was developed based on literature and combined with input from specialists of laboratory medicine and GPs. Literature searches were performed using Scopus™ (Elsevier BV) and Medline/PubMed (US National Library of Medicine). Search strings were ‘patient safety’, ‘primary care’, ‘general practitioner’, ‘point of care testing’, ‘point of care tests’, ‘blood glucose test’, ‘nitrite test’ and ‘haemoglobin test’. Important safety and quality aspects in the questionnaire included i) training of users, ii) intake and storage of test materials, calibration and maintenance of the equipment during the pre-analytical phase, iii) hygiene procedures before collecting samples, test performance, registration of results during the analytical phase and iv) actions based on test results during the post-analytical phase. Table 1 provides an overview of the key elements investigated in this study. All questions were close-ended, with in some cases the opportunity to add comments. For several questions, respondents could choose more than one answer. A sample questionnaire is available as Additional file 1.

**Table 1:** **Overview of the key elements for managing patient safety aspects related to POCT investigated in this study**

**Key elements**	**Description**
Training of users	protocol available as part of the quality system,
	users must be trained, certified
	periodical refresher courses
Pre-analytical phase	check control measures upon delivery of POC tests:
	-storage condition
	-packaging undamaged
	-expiry dates
	check control measures before using POC tests:
	-instruct the patient
	-read instructions for use or own written protocol
	-check storage condition
	-packaging undamaged
	-expiry dates
	-regular maintenance and calibration of POC test meters
Analytical phase	perform patient identification
	take the appropriate hygienic measures (e.g. washing hands )
	correct sample handling
Post-analytical phase	record test results
	take control measures and action in case device failure, such as:
	-refer a patient or sent the sample to a laboratory for testing
	-contact the manufacturer of the POC test
	-check the expiry date of the POC test
	-repeat the test with an other POC test
	-repeat the test with a new sample
	take control measures and a actions in case the test results conflict with symptoms, such as:
	-refer a patient or sent the sample to a laboratory for testing
	-repeat the test with an other POC test
	-repeat the test with a new sample
	-give life style advice
	prescribe medication

### Study population, data collection and data analyses

Out of 4090 GP practices registered in the national registry of practices in the Netherlands [12] a random sample of 750 GP practices (18%) was drawn, taking into account the distribution of solo practices and duo or group practices. This register, maintained by the Netherlands institute for health services research (NIVEL), contains nearly all self-employed GPs and GPs employed by other GPs in the Netherlands. In 2011, an invitation letter was sent to these GP practices by mail, with information on the aim of the study, and a link to the web-based questionnaire (SurveyMonkey^TM^). GP practices have to have a quality management system according to the Dutch Health Institutions Quality Act [13]. It is assumed that quality management system in GP practices lead to a relatively uniform way of working within one practice. Therefore, per practice, only one web-based questionnaire could be filled out. In the letter, it was clearly stated that the questionnaire should be filled out by one of the professionals within the practice who actually uses POC tests. Participation to the study was voluntary, and all obtained data were processed anonymously, not traceable to the individual GP practices. According to Dutch Civil Law, Article 7:458, this study did not require ethics approval. To maximize the response a reminder was sent to non-respondents three weeks after the first mailing. Incomplete questionnaires were excluded. Descriptive statistics were used to describe demographic characteristics of the respondents and the information on actual use of POC tests. A chi-square test ( χ^2^) was used to investigate the representativeness of the GP practices, i.e. the differences between the distribution of practice types within the random sample and within the group of GP practices that returned the questionnaire. The difference was considered statistically significant, if p < 0.05 ( χ^2^ test). For the analyses the Statistical Package for Social Sciences (SPSS) software (IBM SPSS Statistics 19) was used.

## Results

### Demographic characteristics of the respondents

The random sample of 750 GP practices consisted of 47% solo practices and 53% duo or group practices, which was similar to the total population of GP practices (n = 4090) (46% solo practices, 54% duo or group practices). Out of 750 GP practices, eight GP practices responded that they could not participate in the study because of various reasons, e.g. long-term sickness leave or retirement. Two GP practices responded that they did not want to participate in the study. Of the remaining 740 GP practices, 111 (15%) responded by returning the questionnaire. No significant difference (p = 0.6; χ^2^ test) in distribution of practice types was observed between the random sample of GP practices and the GP practices that returned the questionnaire (50% solo and 50% duo or group practices). Characteristics of the respondents are summarized in Table 2.

**Table 2:** **Characteristics of respondents (n = 111)**

**Demographic variables**		**n**	**(%)**
Function	general practitioner	67	60
	practice assistant	39	35
	practice nurse	2	2
	Other^*^	3	3
Type of practice	Solo	45	41
	Solo, NHG^+^ accredited	11	10
	Duo or group	36	32
	Duo or group, NHG^+^ accredited	19	17

*Pharmaceutical assistant, practice assistant as well as practice nurse; or pharmaceutical quality manager as well as practice assistant.

^+^The Dutch College of General Practitioners (NHG).

Sixty percent of the questionnaires were completed by GPs, 40% by GP assistants or practice nurses (Table 2). Not all questionnaires were used in further analyses due to incomplete answers relating to specific tests. Eighty-six out of 109 (78%) respondents using blood glucose test, 97 out of 105 (87%) respondents using nitrite tests and 45 out of 50 (90%) respondents using haemoglobin tests completed all questions relating to the specific tests (Table 3).

**Table 3:** **Use of POC test in GP practices (n = 111) and completed questionnaires**

	**Number of GP practices:**			
	**Using a POC test**		**Completed the questionnaires**	
**POCT**	**n**	%*	**n**	**(%)**
Blood glucose test	109	98	86	78
		(*91 and 96*)		
Nitrite test	105	95	97	87
		(*96 and 96*)		
Haemoglobin test	50	45	45	90
		(*55 and 58*)		

*Percentages in *italic* from Hofland [11] and Howick et al. [14].

Number of GP practices using a POCT (%) = (n/111)*100.

Number of GP practices completed the questionnaires (%) = (n/n (Number of GP practices using a POCT)*100.

More than 60% of the respondents had experience with POC tests for more than ten years. The frequency of using POC tests was high; nitrite tests were used on a daily basis by 95% of the respondents, blood glucose meters were used daily by 51% and weekly by 41% of the respondents, and haemoglobin tests were used weekly by 69% of the respondents.

### Training of users

In more than 90% of the GP practices, training on using POC tests is given. In most GP practices (>75%) the training was given by professionals working at the practice, such as the GP or practice nurse. Training provided by external professionals, such as the manufacturer of the POC tests or an employee at a clinical laboratory was done in less than 20% of the GP practices. In some of the GP practices (<6%) no training was provided. Refresher courses, however, are hardly ever organized (less than 3%). Even when the test or the instructions for use are modified, less than 30% of the respondents organize a refresher course. Approximately half of the GP practices has a test procedure written specifically for their practice. Only a very low percentage of the respondents (less than 10%) indicate that they read the test procedure written specifically for their practice or the instructions for use before they execute a POC test.

### Pre-analytical phase

**Figure 1:**
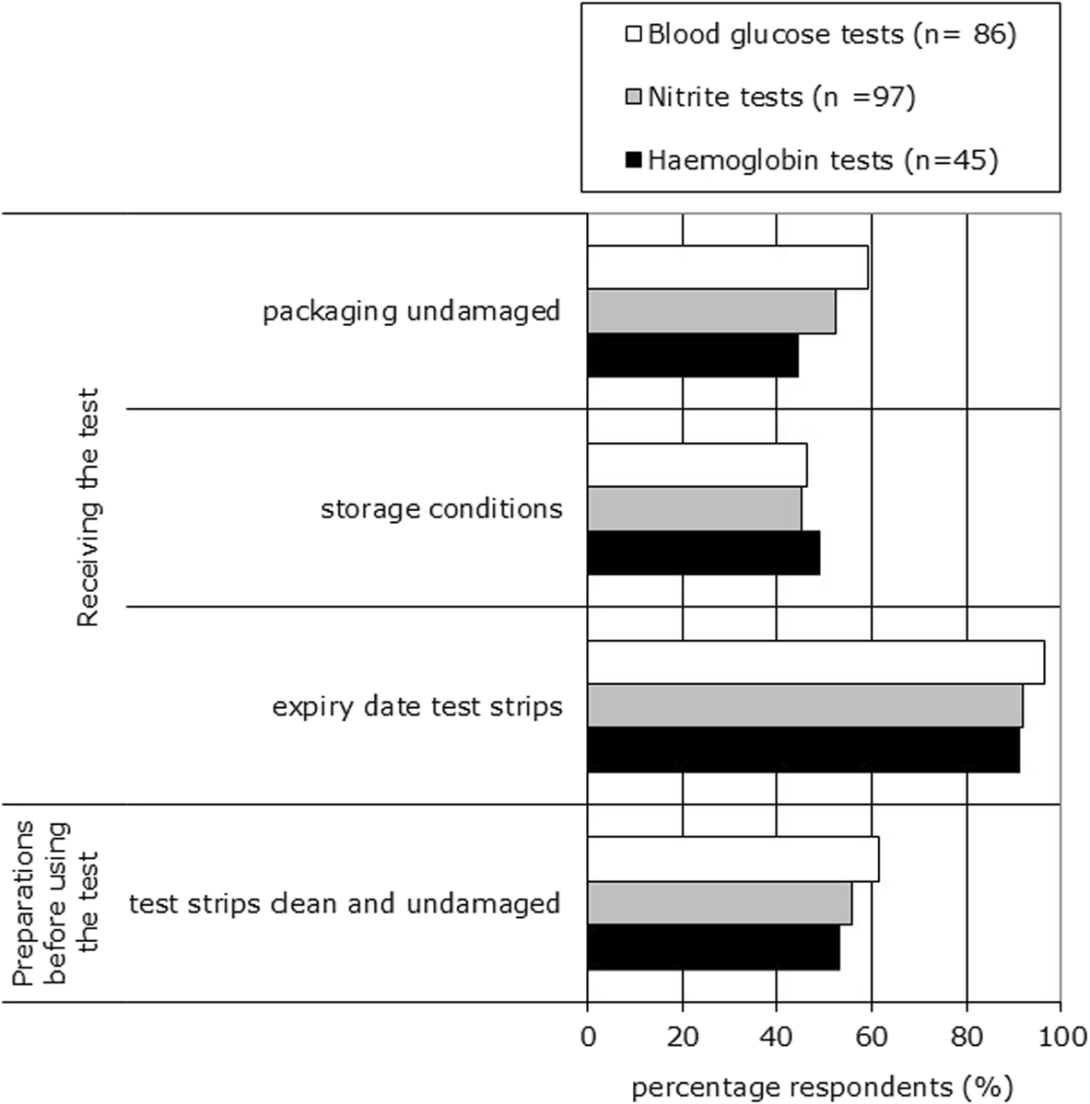
**Pre-analytical phase: control measures for all three POC tests carried out by GP practices.** Respondents could choose more than one answer.

The pre-analytical phase starts as soon as POC tests are being delivered to the GP practices. More than 90% of the respondents check the expiry date of the test strips (for all three tests) as soon as these materials are delivered to the GP’s office. However, they pay less attention (less than 65% for all three tests) to other important aspects, such as storage conditions, possible damage to the packaging and possible unclean and damaged test strips (see Figure 1).

Fifty-one percent of the respondents using blood glucose meters and 44% of the respondents using haemoglobin meters check before use whether the codes of the test strip and the meter correspond. Some respondents answered that checking the code of the test strips and meters is not necessary because of new types of meters. A small percentage of respondents (20%-26%) using blood glucose and haemoglobin meters check whether the meter is calibrated or generally maintained.

### Analytical phase

The majority of the respondents provides instructions to the patients (e.g. on how to collect a urine sample) before they use the tests. More than 75% of the respondents pay attention to patient identification. Less attention is paid to other safety aspects during the analytical phase.

**Figure 2:**
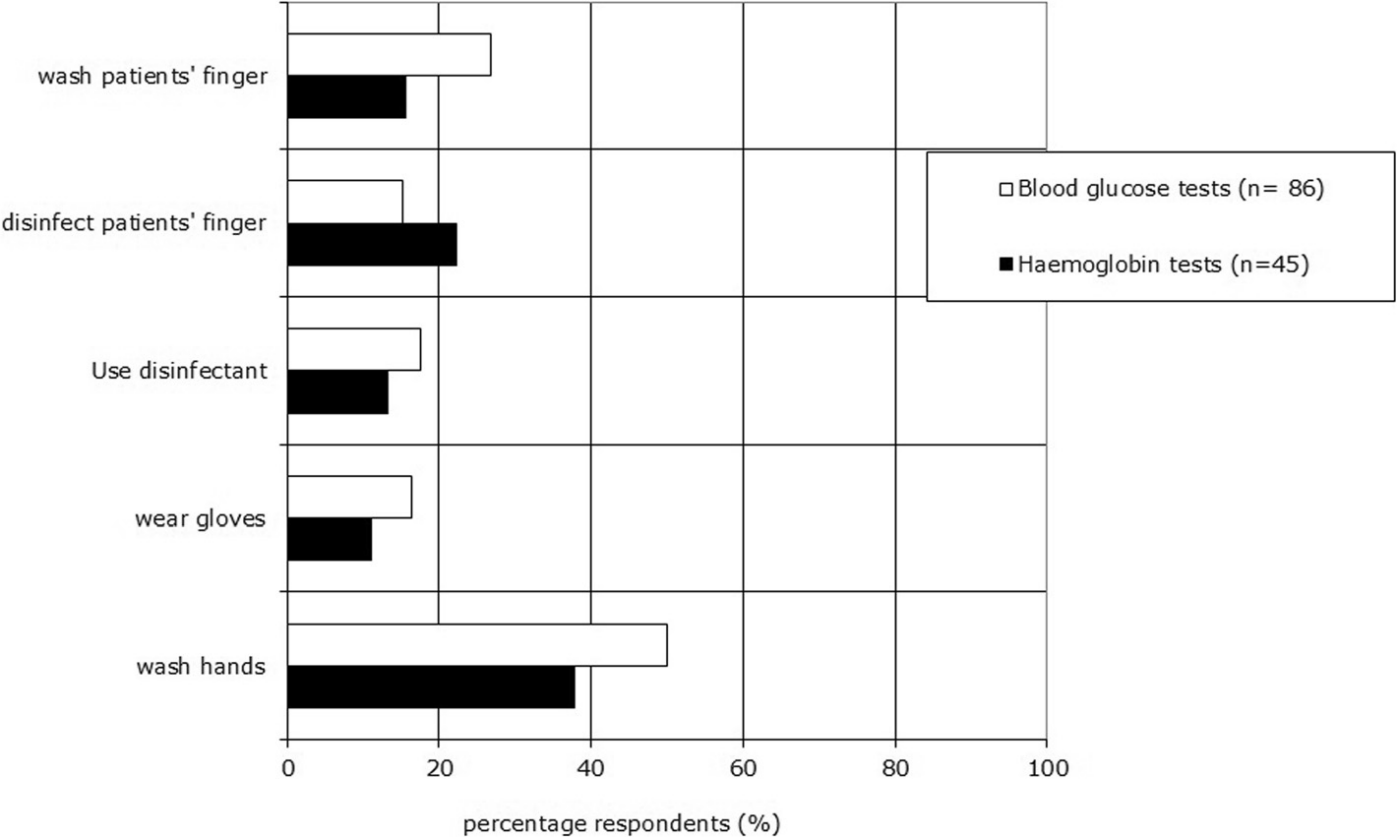
**Analytical phase: hygienic measures.** Respondents could choose more than one answer. Twelve percent of the blood glucose test users and nine percent of the haemoglobin test users answered that they wash their hands and wear gloves before taking a blood sample.

About half of the respondents using blood glucose tests and only 38% of the respondents using haemoglobin tests takes hygienic measures, such as washing their own hands before taking a blood sample. Less than 20% of the respondents indicated that they wear gloves. Washing/disinfecting the patients’ finger before blood sampling is performed by less than half of the respondents (see Figure 2).

More than half of the respondents using blood glucose or haemoglobin tests pay attention to removing the first blood drop and not squeezing the finger to obtain a blood sample. Six percent of the blood glucose meter users and 9% of the haemoglobin meter users do not give attention to either of these aspects. To obtain reliable test results, the test area of the test strips for blood glucose and haemoglobin must be completely filled. Many users, 88% for blood glucose and 82% for haemoglobin, indicated that they pay attention to this aspect.

Most respondents (87%) indicated that only first morning urine samples were accepted. A small number of respondents (13%) accepts all types of urine samples, for instance when a patient has serious complaints matching a suspected urinary tract infection. In these cases, the serious clinical symptoms outweigh a poor quality of the urine sample or negative nitrite levels. Some respondents reported that they perform additional tests if the results of the nitrite tests are negative. Additional testing includes repeating the nitrite test or performing a dipslide test^a^.

### Post-analytical phase

Since results of laboratory tests need to be integrated in the patients’ medical records, it was investigated how this is carried out for POC test results. A vast majority of the respondents enter the test results manually in their electronic health record (EHR) system (blood glucose tests 92%, nitrite test 89% and haemoglobin test 87%). Some respondents record the data via an electronic link between the device and their EHR system (blood glucose tests 12%, nitrite test 11% and haemoglobin test 13%). Less than 6% of blood glucose test users and nitrite test users record the test results manually in the non-electronic medical record of the patient or on a specific paper form for test results.

#### Check and action after device failure

**Figure 3:**
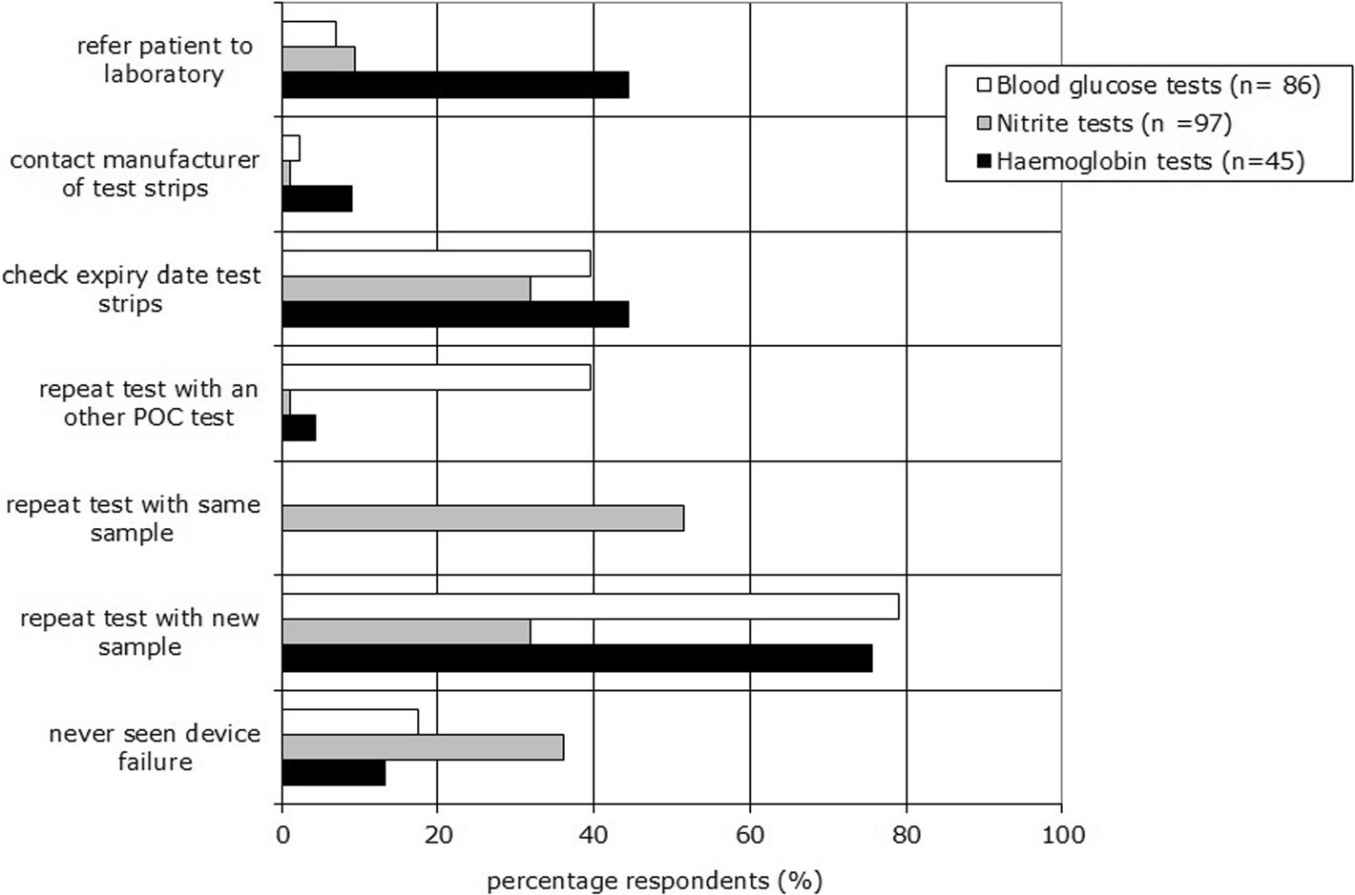
**Post-analytical phase: control measures and actions in case of device failure for all three POC tests carried out by GP practices.** Respondents could choose more than one answer.

More than 75% of the blood glucose test users and of haemoglobin test users indicated they repeat the test with a new sample if device failure occurs. Of the nitrite test users, 32% repeat the test with a new urine sample, whereas 52% indicated they repeat the test with the same urine sample. A very low percentage of the respondents (less than 10%) contact the manufacturer of the device in case of device failure. Also ‘referring a patient to a laboratory’ was an answer not often chosen by the users of blood glucose meters and nitrite tests (less than 10%). For the haemoglobin test users this was 44%. Some respondents indicated that they never experience a device failure: for blood glucose tests 17%, for haemoglobin tests 13%, and for nitrite test 36% (see Figure 3).

In case of a device failure for the blood tests, a relatively small number of the respondents pay attention to extra control measures such as:

- Checking the meter (56% blood glucose test, 38% haemoglobin test).
- Checking whether the test strip was inserted correctly (49% blood glucose test, 47% haemoglobin test).
- Checking whether the test area of the test strips was completely filled (55% blood glucose test, 58% haemoglobin test).
- Collecting a blood sample by venipuncture and sending it to a laboratory (blood glucose tests 4% and haemoglobin tests 18%).
- Reading the instruction for use to identify the error and find solutions for the problem (less than 3% of the blood test users).

For the nitrite tests, a relatively small number of the respondents pay attention to extra control measures such as:

- Sending the urine samples to a laboratory (16%).
- Examining the sediment of the urine sample (8%).
- Taking a dipslide sample to determine if there was a bacterial infection (5%).

#### Checks and actions after conflicting test outcomes for blood glucose tests

A high percentage (98%) of the respondents using blood glucose tests indicated that high blood glucose levels always lead to action, even when no symptoms are observed. In addition, more than half of them take action if the blood glucose levels are too low and no symptoms are observed.

**Figure 4:**
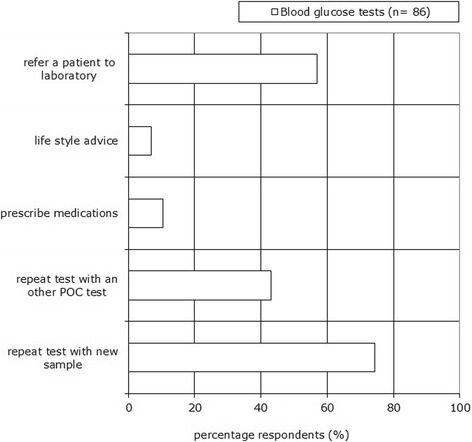
**Post-analytical phase: control measures and actions in case blood glucose test results conflict with symptoms.** Respondents could choose more than one answer.

Of the respondents, 74% repeat the test with a new sample and 57% refer the patient to a laboratory when test results and symptoms conflict. Less than 50% of the respondents repeated the test with another POC test. And less than 20% of the respondents using blood glucose test reported that they prescribe medication or give life style advice if test results conflict with symptoms (see Figure 4).

#### Checks and actions after conflicting test outcomes for nitrite tests

Most respondents take extra measures, such as performing a dipslide test or repeating the test, when they observe symptoms of urinary tract infection while nitrite test outcomes are negative. In addition to these actions, almost a quarter of the respondents prescribe antibiotics when nitrite test results are negative.

Respondents using nitrite test were also asked what kind of action they take when nitrite test results are positive. As expected, most respondents (95%) prescribe antibiotics and 55% give life style advice. In some cases also a dipslide test (16%) is performed, for example when a relapse occurs.

#### Checks and actions after test outcomes for haemoglobin tests

Only a small number (20%) of GP practices indicated they make inquiries about the patient’s diet if haemoglobin levels deviate from reference values. More than half of the GP practices immediately prescribe or adjust the patient’s medication. Sixty percent of the GP practices refer patients to a medical specialist if haemoglobin is too low.

## Discussion

In this exploratory study it was investigated how patient safety aspects of three POC tests are managed in Dutch GP practices. The percentages of GP practices using these tests (blood glucose: 98%, nitrite: 95% and haemoglobin: 45%) in this study are comparable with the results from two other studies among Dutch GP practices [11,14] and the frequency of use of the three POC tests was high. Results of our study show that training is provided in almost all the GP practices. However, in most practices training is provided by professionals working at the practice (second hand). Whether training is provided directly by a manufacturer of the POC test to the user or second hand (e.g. a GP) may have impact on the protocol use and safety outcomes. Furthermore, refresher courses are hardly ever organized and protocols written specifically for the practice are not always available. Errors in using the test may be introduced gradually and go unnoticed if experiences and problems are not periodically discussed and evaluated [15]. Regular training would help to mitigate the risk of unnoticed errors. In a study in three different hospitals in the UK, the importance of training and adherence to protocols by operators of POC tests was emphasized [2], because most errors were related to the fact that the operator was unable to use the POC test correctly. Because of the hospital setting, in case of ambiguous results a sample was sent to the central laboratory for analysis. Therefore, erroneous results from POC test did not lead to any adverse clinical outcomes [2]. However, in GP practices there is no instantly available laboratory service and impact of errors on the clinical outcome may be larger Results from two studies on training of device operators in Australia show that with the appropriate training and support, GP practices can achieve similar levels of safety and accuracy as that found in laboratories [16,17]. Moreover, the Netherlands Society for Clinical Chemistry and Laboratory Medicine recommends for use of POC blood glucose tests in hospitals that (1) users must have a protocol available as part of the quality system, (2) that they must be trained and pass an exam, and (3) that a periodical refresher course should be introduced [3-5]. These recommendations can be considered relevant for all users of POC tests and all types of POC tests.

Respondents of our study paid less attention to storage condition of the POC tests. Environmental influences, such as exposure to light, air and humidity have a negative effect on the stability of the chemicals on the test strips and should be taken into account [18]. Furthermore, some respondents stated that checking whether the test strips and meter correspond is not necessary for new types of meters. There are, however, several cases of incorrect blood glucose levels obtained with test strips not corresponding with the meters [19]. Checking if the test strips and meter correspond is an important control measure before using POC tests. Although most respondents of this study had considerable experience in using POC tests, the majority using blood glucose and haemoglobin meters did not check whether the meter was calibrated or generally maintained. Neglecting calibration and maintenance of equipment can cause erroneous outcomes of the measurement [10,20,21]. Hygienic measures, such as washing or disinfect hands and wear gloves when taking a blood sample was done by just a few of the respondents. These measures are important to protect the patient and the professional against transfer of infectious materials and to exclude interference of other substances. Therefore the professional should wash hands or use disinfectants, and wear gloves [22,23]. In recent years, several reports describe a lack of hygiene leading to erroneous test results [20,24]. Contamination with sugar, orange juice, et cetera, influences results and may subsequently lead to wrong decisions on e.g. administering insulin.

Most of the respondents take the correct actions such as instructing the patient, collecting the samples, and conducting the tests. Several respondents indicated that they repeat the test if device failure occurs. Other answering options, such as contacting the manufacturer or referring a patient to a laboratory, were selected less often. As part of European legislation on in vitro diagnostic medical devices, which is applicable for POC devices, a manufacturer should have a post market surveillance procedure which contains both active (e.g., customer satisfaction surveys) and passive (e.g., complaints registration) elements for gathering the experiences of users of the device [25]. Surveillance findings can lead to the re-assessment of a risk, improvements in the design of the device, or amendments in the instructions for use. However, if the manufacturer is not informed, the device or instructions for use will not be improved.

The majority of the respondents using the blood glucose tests and haemoglobin tests take appropriate actions after they observe a conflict between the test result and the symptoms. Just a small number of respondents using haemoglobin tests reported that they make inquiries about the patient’s diet if haemoglobin level is low. This is remarkable, since a well-known possible cause if anaemia could be an unbalanced diet, which can be managed relatively well [26].

For the nitrite test, the GP practices in our study are less precise when test results conflict with symptoms. A high percentage of respondents prescribed antibiotics when nitrite test results were negative and symptoms were positive. If the result of the nitrite test is negative, whereas the clinical signs point towards an infection, a dipslide test is recommended by the national guideline of NHG [7]. In addition, the guideline recommends taking a dipslide test or a laboratory test to detect complicated infections if the urine sample is from a male, or to detect antimicrobial resistance. However, this guideline also provides the option to prescribe antibiotics for a defined patient population if symptoms indicate a urinary tract infection, even without performing a test. This may partly explain the high percentage of respondents prescribing antibiotics when nitrite test results were negative and symptoms were positive. It should be kept in mind, however, that antibiotic resistance is a worldwide problem [27], and therefore GPs always have to carefully weigh the risks and benefits of prescribing antibiotics.

### Limitation of the study

From the 750 GP practices, 111(15%) participated in the study. Although this is a rather low response, this is not unusual. Results from other studies among general practitioners show that this group is difficult to observe [28-31]. For example, in an online survey among all GPs in Australia the response rate was less than 0.1% [29] and in a study among 600 Dutch GPs 157 (26.2%) responded [30]. Lack of time or less interest in the topic of a study seem to be the reasons for the low response rate on surveys among general practitioners [32]. Besides the low response rate, the results may be biased by socially desired responses. Furthermore, a selective response may have occurred, in that GPs interested in this topic, and thus paying attention to using POC tests, returned the questionnaire. One person per practice could fill out the questionnaire, and within one practice differences between persons may occur. All these aspects may contribute to a overestimation or underestimation of some of the results when extrapolating these to all GP practices in the Netherlands. However, this is the first study to systematically evaluate safety aspects of POC test use, with an emphasis on the use by GP practices in the Netherlands. The results of this study gives a good indication on how GP practices, included in this study, manage patients safety aspects related to POC tests. And although, questions were based on three POC tests, it is very likely that the results from this study are also applicable for the management of patient safety aspects related to other types of POC tests.

## Conclusions

The results of this study demonstrate how Dutch GP practices manage patient safety aspects related to POC tests. Although we found that some of the key elements were adopted by most GP practices, also shortcomings were observed in important aspects, such as hygienic measures, training, quality control measures and actions based on test results. We conclude that there is much room for improvement, and that our findings call for further research. It would be useful to investigate how often shortcomings have led to adverse events. To encourage proper use of POC tests in general practices, a national POC test guideline, dedicated to primary care and in line with ISO standards, should be introduced. A guideline should address the issues described in this paper and include patient safety, maintenance, standard operating procedures and quality management. The implementation and adherence to such guideline should be incorporated into the national accreditation system for general practices.

## Endnote

^a^A dipslide test is a plastic paddle coated on both sides with agar media. Dipslides are inoculated by dipping the agar-coated slides in the urine sample, where they are incubated at 37°C for 18–24 hours. After incubation the amount of bacterial colonies is determined and compared to a model chart provided by the manufacturer of the dipslide device [15].

## Additional file

Additional file 1:
**Questionnaire.**
