# Supplementary material for: Results of a survey among GP practices on how they manage patient safety aspects related to point-of-care testing in every day practice
Source: BMC Fam Pract. 2015 Feb 5;16:9. doi: 10.1186/s12875-014-0217-2 (PMC4332919; doi:10.1186/s12875-014-0217-2)
Supplement: Additional file 1: — Questionnaire. [file 12875_2014_217_MOESM1_ESM.docx]

**Questionnaire**

**Managing patient safety aspects related to point-of-care testing in every day practice**

1. What is your position?

 General practitioner

 Practice assistant

 Practice nurse

 Other: …….…

2. How many years have you been in your current position?

 0 – 5 years

 6 – 10 years

 11 – 15 years

 16 years or more

3. In what kind of practice do you work?

 Solo practice

 Solo practice, accredited by the Dutch College of General Practitioners (NHG)

 Duo or group practice

 Duo or group practice, accredited by the NHG

**Questions related to the use of blood glucose POC tests**

4. Do you use blood glucose tests in your practice?

 Yes

 No, go to question 18.

5. Since when are blood glucose tests used in your practice?

Since |__|__||__|__||__|__||__|__| (ddmmyyyy)

6. How often do you use a blood glucose test in your practice?

 Daily

 Weekly

 Monthly

 Yearly

7. Is there a test procedure for the blood glucose tests, which has been written specifically for your practice?

 Yes

 No

8. Who gives instruction to the employees on how to use the blood glucose tests?

 General practitioner

 Manufacturer

 Practice assistant

 Practice nurse

- - No instructions are given (go to question 10)
  - Other:……………………….

9. Is the instruction completed with an exam?

 Yes, we register who have passed the exam

 Yes, however we do not register who have passed the exam

 No

10. Do you organize refresher courses for the use of the blood glucose test?

 Yes, …… times a year

 Yes, only if the tests or instructions for use were changed

- - No
  - Other:……………………….

11. Which control measures do you take when receiving new blood glucose test materials? (Multiple answers are possible)

 Check the expiry data of test strips

 Check storage conditions of test strips (e.g. temperature of storage space)

 Completeness of the test equipment

 Check if the codes of the meter and test strips correspond

 Check if the sealing of the test strips is undamaged

 None of the above, I may assume that the POC test materials are of good quality

 Other:……..

12. Which preparations do you take before performing a blood glucose test?

(Multiple answers are possible)

 Give instruction to the patient (e.g. dietary advice)

 Check expiry date of test strips

 Check storage conditions of test strips (e.g. temperature of storage location)

 Check latest moment of calibration

 Check latest moment of maintenance

- - Check if test strips are clean and undamaged
  - Completeness of the test equipment
  - Read the instructions for use
  - Read the test procedure, written specifically for our practice

 Other:……..

13. Which aspect(s) do you consider when taking a blood sample from a finger prick and performing the test? (Multiple answers are possible)

 Patient identification

- - Washing your hands
  - Wearing gloves

 Use of disinfectant

- - Washing the finger of the patient
  - Disinfecting the finger of the patient
  - Removing first drop of blood
  - Preventing pushing blood from the finger
  - Filling the test area of the test strip completely

 Other:……..

14. How do you record the test results? (Multiple answers are possible)

 Manually, in the paper dossier of the patient

 Manually, on a specific paper form for test results

 Manually, in an electronic health record (EHR) system

- - Manually, as free text on the E and/or P rules of the S,O, E or P rules* of the EHR system
  - Automatically, via an electronic link between device and registry system
  - Test results are not recorded
  - I do not know

* S- subjective: reason for contact, O-objective: physical exam, E- evaluation: diagnose, P- treatment plan

15. Which action(s) do you take when the blood glucose meter shows an error?

(Multiple answers are possible)

 I never get errors

 Repeat the test

 Check whether the test area of the test strip is completely filled with blood

 Check whether the test strip is inserted correctly into the meter

 Check the meter (e.g. battery)

 Check expiry date of the test strips

 Contact the manufacturer of the test

 Use another meter

 Refer the patient to a laboratory

 Collect a blood sample via venipuncture and send the sample to a laboratory

 I do not take actions

 Other:……..

16. Which action(s) do you take when test results conflict with the symptoms?

(Multiple answers are possible

 Check the meter (e.g. battery)

 Repeat the test

 Use another meter

 Collect a blood sample via venipuncture and send the sample to a laboratory

 Refer the patient to a laboratory

 Refer the patient to a specialist

 Life style advice (e.g. diet)

 Prescribe or adjust medication

 I do not take actions

 Other:……..

17. Which test results and symptoms lead to action?

(Multiple answers are possible

 Glucose levels high, no symptoms

 Glucose levels high, symptoms

 Glucose levels normal, no symptoms

 Glucose levels normal, symptoms

 Glucose levels low, no symptoms

 Glucose levels low, symptoms

- - I do not know

**Questions related to the use of Nitrite POC tests**

18. Do you use nitrite tests in your practice?

 Yes

 No, go to question 33

19. Since when are nitrite tests used in your practice?

Since |__|__|__|__|__|__|__|__| (ddmmyyyy)

20. How often do you use a nitrite test in your practice?

 Daily

 Weekly

 Monthly

 Yearly

21. Is there a test procedure for the nitrite tests written specifically for your practice?

 Yes

 No

22. Who gives instruction to the employees on how to use the nitrite tests?

 General practitioner

 Manufacturer

 Practice assistant

 Practice nurse

- - No instruction is given (go to question 24)
  - Other:……………………….

23. Is the instruction completed with an exam?

 Yes, we register who have passed the exam

 Yes, however we do not register who have passed the exam

 No

24. Do you organize refresher courses for the use of the nitrite test?

 Yes, …… times a year

 Yes, only if the tests or instructions for use are changed

- - No
  - Other:

25. Which control measures do you take when receiving the new nitrite test materials? (Multiple answers are possible)

 Check the expiry data of test strips

 Check storage conditions of test strips (e.g. temperature of storage location)

 Check if the sealing of the test strips is undamaged

 None of the above, I may assume that the POC test materials are of good quality

 Other:……..

26. Which preparations do you take before performing a nitrite test?

(Multiple answers are possible)

 Give instruction to the patient (e.g. on how to collect a urine sample)

 Check expiry date of test strips

 Check storage conditions of test strips

 Check if test strips are clean and undamaged

 Read the instructions for use

 Read test procedure written specifically for our practice

 Other,……..

27. What kinds of urine samples do you accept when a patient brings his/her urine sample from home? (Multiple answers are possible)

 First morning urine samples

- - First morning samples that are collected at a maximum of two hours before actual testing.
  - All types of urine samples
  - I do not know
  - Other,……..

28. Which aspect(s) do you consider when performing a Nitrite test? (Multiple answers are possible)

 Patient identification

 Other,……..

29. How do you record the test results? (Multiple answers are possible)

 Manually, in the paper dossier of the patient

 Manually, on a specific paper form for test results

 Manually, in the Netherlands information Network of General Practice (LINH) database

- - Manually, as free text on the E and/or P rules of the S,O, E or P rules* of the LINH database
  - Automatically, via an electronic link between device and registry system
  - Test results are not recorded
  - I do not know

* S- subjective: reason for contact, O-objective: physical exam, E- evaluation: diagnose, P- treatment plan

30. Which action(s) do you take when the nitrite test strip is unreadable (device failure)? (Multiple answers are possible)

 Test strips are always readable

 Repeat the test with the same urine sample

- - Check the storage condition of the test strips
  - Check expiry date of the test strips
  - Collect new urine sample
  - Use another batch of test strips
  - Contact the manufacturer of the test

 Refer a patient to a specialist

 Refer a patient to a laboratory

 Send the sample to a laboratory

 I do not take actions

 Other:……..

31. Which action(s) do you take when test results conflict with the symptoms?

(Multiple answers are possible)

 Check urine sample with other point-of-care test

 Test urine sample with dip slide test

- - Send the urine sample to a laboratory
  - Collect new urine sample

 Refer the patient to a laboratory

 Life style advice

 Prescribe antibiotics

 Other:……..

32. Which action(s) do you take when test results point toward a urinary tract infection? (Multiple answers are possible)

 Test the urine sample with another point-of-care test

 Test the urine sample with a dip slide test

- - Send the urine sample to a laboratory
  - Collect new urine sample

 Refer the patient to a laboratory

 Life style advice

 Prescribe antibiotics

 Other:……..

**Questions related to the use of haemoglobin POC tests**

33. Do you use haemoglobin tests in your practice?

 Yes

 No.

34. Since when are haemoglobin tests being used in your practice?

Since |__|__|__|__|__|__|__|__| (ddmmyyyy)

35. How often do you use a haemoglobin tests in your practice?

 Daily

 Weekly

 Monthly

 Yearly

36. Is there a test procedure for the haemoglobin tests written specifically for your practice?

 Yes

 No

37. Who gives instruction to the employees on how to use the haemoglobin tests?

 General practitioner

 Manufacturer

 Practice assistant

 Practice nurse

- - No instructions are given (go to question 10)
  - Other:……………………….

38. Is the instruction completed with an exam?

 Yes, we register who have passed the exam

 Yes, however we do not register who have passed the exam

 No

39. Do you organize refresher courses for the use of the haemoglobin test?

 Yes, …… times a year

 Yes, only if the tests or instructions for use were changed

- - No
  - Other:……………………….

40. Which control measures do you take when receiving new haemoglobin test materials? (Multiple answers are possible)

 Check the expiry date of test strips

 Check storage conditions of test strips (e.g. temperature of storage location)

 Completeness of the test equipment

 Check if the codes of the meter and test strips correspond

 Check if the sealing of the test strips is undamaged

 None of the above, I may assume that the POC test materials are of good quality

 Other:……..

41. Which preparations do you take before performing a haemoglobin test?

(Multiple answers are possible)

 Give instruction to the patient (e.g. dietary advice)

 Check expiry date of test strips

 Check storage conditions of test strips (e.g. temperature of storage location)

 Check latest moment of calibration

 Check latest moment of maintenance

- - Check if test strips are clean and undamaged
  - Check completeness of the test equipment
  - Read the instruction for use
  - Read test procedure, written specifically for our practice

 Other:……..

42. Which aspect(s) do you consider when taking a blood sample from a finger prick and performing the test? (Multiple answers are possible)

 Patient identification

- - Washing your hands
  - Wearing gloves

 Use of disinfectant

- - Washing the finger of the patient
  - Disinfecting the finger of the patient
  - Removing first drop of blood
  - Preventing pushing blood from the finger
  - Filling the test area of the test strip completely

 Other:……..

43. How do you record the test results? (Multiple answers are possible)

 Manually, in the paper dossier of the patient

 Manually, on a specific paper form for test results

 Manually, in the Netherlands information Network of General Practice (LINH) database

- - Manually, as free text on the E and/or P rules of the S,O, E or P rules* of the LINH database
  - Automatically, via an electronic link between device and registry system
  - Test results are not recorded
  - I do not know

* S- subjective: reason for contact, O-objective: physical exam, E- evaluation: diagnose, P- treatment plan

44. Which action do you take when the haemoglobin meter shows an error?

(Multiple answers are possible)

 I never get errors

 Repeat the test

 Check whether the test area of the test strip is completely filled with blood

 Check whether the test strip is inserted correctly into the meter

 Check the meter (e.g. battery)

 Check expiry date of the test strips

 Contact the manufacturer of the test

 Use another meter

 Refer the patient to a laboratory

 Collect a blood sample via venipuncture and send the sample to a laboratory

 I do not take actions

 Other:……..

45. Which action do you take when test results show abnormal results? (Multiple answers are possible

 Check the meter

 Repeat the test

 Use another meter

 Collect a blood sample via venipuncture and send the sample to a laboratory

 Refer the patient to a laboratory

 Refer the patient to a specialist

- - Collect a new urine sample

 Inquire about the patient’s diet

 Prescribe or adjust medication

 I do not take actions

 Other:……..

End of the questionnaire!
